# Mitochondrial DNA variation in sudden cardiac death: a population-based study

**DOI:** 10.1007/s00414-019-02091-4

**Published:** 2019-05-31

**Authors:** Laura Kytövuori, Juhani Junttila, Heikki Huikuri, Sirkka Keinänen-Kiukaanniemi, Kari Majamaa, Mika H. Martikainen

**Affiliations:** 1 Research Unit of Clinical Neuroscience, University of Oulu, PO Box 5000, 90014 Oulu, Finland; 2 Medical Research Center Oulu, Oulu University Hospital and University of Oulu, Oulu, Finland; 3 Department of Neurology, Oulu University Hospital, PO Box 20, 90029 Oulu, Finland; 4 Research Unit of Internal Medicine, University of Oulu, PO Box 5000, 90014 Oulu, Finland; 5 Center for Life Course Health Research, University of Oulu, PO Box 5000, 90014 Oulu, Finland; 6 Healthcare and Social Services of Selänne, Pyhäjärvi, Finland; 7 Division of Clinical Neurosciences, University of Turku and Turku University Hospital, Kiinamyllynkatu 4-8, 20520 Turku, Finland

**Keywords:** Mitochondrial disease, Mitochondrial DNA, Mitochondrial haplogroups, Non-ischaemic, Sudden cardiac death

## Abstract

Cardiomyopathy and cardiac conduction defects are common manifestations of mitochondrial disease. Previous studies suggest that clinically asymptomatic individuals harbouring pathogenic mitochondrial DNA (mtDNA) mutations in the cardiac muscle may have sudden cardiac death (SCD) as the first manifestation of mitochondrial disease. We investigated the contribution of pathogenic mtDNA point mutations and mtDNA haplogroups in cardiac muscle in a cohort of 280 Finnish subjects that had died from non-ischaemic SCD with the median age of death at 59 years and in 537 population controls. We did not find any common or novel pathogenic mutations, but the frequency of haplogroup H1 was higher in the SCD subjects than that in 537 population controls (odds ratio: 1.76, confidence interval 95%: 1.02–3.04). We conclude that, at the population level, pathogenic point mutations in mtDNA do not contribute to non-ischaemic SCD, but natural variation may modify the risk.

**Electronic supplementary material:**

The online version of this article (10.1007/s00414-019-02091-4) contains supplementary material, which is available to authorized users.

## Introduction

Sudden cardiac death (SCD) accounts for 15–20% of all deaths, and the population rates range between 50 and 100/100,000 [1]. In the Finnish population, the annual incidence of SCD has been estimated to be 56.9/100,000 [2]. Coronary artery disease (CAD) is the most common (~ 80%) cause of SCD; cardiomyopathy (CM) and fibrotic CM explain about a third of non-ischaemic SCD events [2]. Among younger SCD subjects, however, monogenic disease leading to cardiomyopathy and various arrhythmias plays a major role [3]. Non-ischaemic SCD, particularly among young individuals, is etiologically multifactorial, and in many instances, no single causal factor can be confirmed with certainty [4].

Mitochondrial diseases can manifest as cardiomyopathy, conduction defects and even SCD [5]. The m.3243A > G point mutation in the *MT-TL1* gene is the most common pathogenic mtDNA mutation and causes variable clinical manifestations [6]. Carriers of the m.3243A > G mutation have an increased risk of death, and sudden death resulting from cardiac complications is not uncommon among them [7, 8]. Importantly, clinically asymptomatic carriers of m.3243A > G may harbour the mutation with a high portion (heteroplasmy) in the cardiac tissue, predisposing them to failure of heart energy production and SCD [8]. Cardiac manifestations seem to be particularly prevalent in association with certain other mtDNA mutations, such as m.3260A > G [9, 10].

Sequence variation in mtDNA may be nonneutral, as mtDNA haplogroups have been associated with disease risks and functional differences have been observed in oxidative phosphorylation (OXPHOS) and production of reactive oxygen species [11–13]. Specific mtDNA haplogroups have been associated with many traits or diseases such as longevity [14], diabetes mellitus [15], obesity [16] and Parkinson’s disease [17]. Despite the paramount role of mitochondrial energy production in cardiac health, there are no previous studies on the role of mtDNA mutations and haplogroups in SCD in population. Therefore, we decided to investigate the frequency of pathogenic mtDNA mutations and that of mtDNA haplogroups in a large, population-based autopsy cohort of Finnish subjects that died from non-ishaemic SCD.

## Subjects and methods

### Study population

Study subjects consisted of persons who had died outside hospital in 2006–2012. Here, SCD was defined as a witnessed cardiac death within 6 h of the onset of symptoms, or within 24 h since the last time the victim was seen alive in good health. Autopsies were carried out at the Department of Forensic Medicine of the University of Oulu. The cardiac cause of death was confirmed in the autopsy and non-cardiac sudden deaths were excluded, as were individuals who had a previous diagnosis of CAD, or who were diagnosed with CAD based on autopsy findings. There were altogether 388 non-ischaemic SCDs [18]. The medical data were collected from the electronic medical records, postmortem examination reports and from questionnaires filled out by the closest relatives of the study subjects [18]. Patients diagnosed with alcoholic cardiomyopathy or infectious heart diseases were excluded from the analyses leaving 280 SCDs that were included in the study. The median age at death was 59 years (ranged from 1 to 90 years) (Table 1). Most of the subjects were diagnosed with cardiomegaly (ICD-10: I51.7) or hypertensive heart disease with or without heart failure (I11.0, I11.9, I13.2). The flowchart of study subject identification and genetic investigations is shown in the supplementary figure.

**Table 1:** Causes of the sudden cardiac death (SCD) among 280 subjects deceased in 2006–2012 in Northern Osthrobothnia, Finland

	ICD-10	Young SCDs*	Men	Women	All SCDs	Men	Women
*N*		91	74	17	280	204	76
Age range (years)		20–55	20–55	26–55	1–90		
Diagnosis *N* (%)							
Cardiomegaly	I51.7	40 (44)	35 (47.3)	5 (29.4)	110 (39.3)	85 (41.7)	25 (32.9)
Hypertensive heart disease	I11.0, I11.9, I13.2	29 (31.9)	23 (31.1)	6 (35.3)	124 (44.3)	87 (42.6)	37 (48.7)
Acute myocarditis	I40.9	6 (6.6)	3 (4.1)	3 (17.6)	11 (3.9)	7 (3.4)	4 (5.3)
Myocarditis in other diseases	I41.8	–	–	–	1 (0.4)	–	1 (1.3)
Dilated cardiomyopathy	I42.0	3 (3.3)	3 (4.1)	–	11 (3.9)	10 (4.9)	1 (1.3)
Hypertrophic cardiomyopathy	I42.2	3 (3.3)	3 (4.1)	–	6 (2.1)	5 (2.5)	1 (1.3)
Other/unspecified cardiomyopathy	I42.8, I.42.9	3 (3.3)	2 (2.7)	1 (5.9)	7 (2.5)	5 (2.5)	2 (2.6)
Conductive heart block	I45.8	1 (1.1)	1 (1.4)	–	1 (0.4)	1 (0.5)	–
Ventricular arrhythmia	I47.0	1 (1.1)	–	1 (5.9)	1 (0.4)	–	1 (1.3)
Other/unspecified cardiac arrhythmia	I49.8, I49.9	2 (2.2)	2 (2.7)	–	2 (0.7)	2 (1.0)	–
Myocardial degeneration	I51.5	2 (2.2)	1 (1.4)	1 (5.9)	5 (1.8)	1 (0.5)	4 (5.3)
Unspecified heart disease	I51.9	1 (1.1)	1 (1.4)	–	1 (0.4)	1 (0.5)	–

The controls were residents of the city of Oulu who were born in 1935 [19]. All the 1008 persons, who met the inclusion criteria on 1 October 1990, were invited to the study. Among the 768 participants, 537 persons gave their blood sample that was used to determine mtDNA haplogroup frequencies in the general population.

### Ethical statement

High standard of ethics according to the WMA Declaration of Helsinki was applied in all investigations described in this manuscript. The research project was approved by the Ethics Committee of Oulu University Hospital (EETTMK 44/2002).

### Molecular genetic analyses

The cardiac samples taken at autopsy were stored in paraffin. DNA from paraffin embedded tissue was extracted using QIAamp DNA FFPE Tissue Kit (Qiagen, Hilden, Germany). Because of the paraffin-embedded starting material, the extraction yielded fragmented mtDNA and, therefore, amplification was carried out using secondary polymerase chain reaction (PCR) of 41 overlapping fragments. We initially aimed at sequencing the entire mtDNA, but as the poor quality of FFPE-derived DNA became imminent, we were compelled to target our resources to obtaining the best possible data in the coding region. The fact that no disease-causing mutations have been found in the control region justified this approach. The reactions were done using *Phire Hot Start II* polymerase according to the provided protocols (Thermo Fisher Scientific, Waltham, MA, USA). Primers and detailed reaction conditions are available in supplementary Table 1. PCR products were sequenced using BigDyeTerminator v1.1 cycle sequencing kit (Applied Biosystems, Thermo Fisher Scientific) with 3500xL Genetic Analyzer (Applied Biosystems) in Biocenter Oulu Sequencing Core. DNA from the control blood samples was extracted by using QIAamp DNA Blood Midi Kit, and haplogroups were determined using restriction fragment length polymorphism (Thermo Fisher Scientific, New England Biolabs, Ipswich, MA, USA) as described previously [20].

We determined the common m.3243A > G and m.3260A > G mutations in 280 SCD subjects by sequencing and the entire coding region of mtDNA (m.577-m.16023) was sequenced in a sub-cohort of 91 young subjects (age at death 20–55 years). Because of mtDNA fragmentation, sequencing covered 100% of the coding region of mtDNA in 64 of the 91 samples and a mean of 77% (range, 48–96%) in the remaining 27 samples. Mitochondrial DNA haplogroups could be determined in 78 out of the 91 young victims by using HaploGrep2 [21] with Phylotree 17 [22]. Odds ratios (OR) and 95% confidence intervals (CI) of 95% were calculated for all haplogroups [23, 24].

## Results

The m.3243A > G and m.3260A > G mutations were not detected in the cardiac tissue of 280 subjects that died from SCD. No previously reported or novel pathogenic mutations were found in the mtDNA sequences of 91 young SCD subjects (supplementary Table 2). One variant in rRNA (m.1923C > T), two in tRNA genes (m.5646C > T, m.5840C > T) and nine nonsynonymous variants in protein coding genes (m.4812G > A, m.5164G > A, m.5319A > T, m.7114C > T, m.7706G > A, m.8156G > A, m.8776C > T, m.9333C > T, m.13718G > C) were considered rare as they were present in less than 10 sequences in the MITOMAP database (MITOMAP: A Human Mitochondrial Genome Database. http://www.mitomap.org, 2019). None of the rare variants in the protein coding genes were predicted to be pathogenic as assessed by PredictSNP [25]. The two variants in tRNA genes were benign according to the MitoTIP predictor for tRNA variants [26], and the rRNA variant has previously been found in controls [27].

The frequency of superhaplogroup HV was 56.4% among the SCD subjects while it was 44.7% in the population controls. The two most prevalent haplogroups among SCD subjects were H1 with a frequency of 27% and U5 with a frequency of 24% (Table 2). Other subhaplogroups of H accounted for 19% and other subhaplogroups of U accounted for 5.1% of the subjects. SCD was associated with haplogroup H1 with an OR of 1.76 (CI 95%: 1.02–3.04, *p* = 0.043). ORs for all other haplogroups were not significant.

**Table 2:** Frequencies of the mtDNA haplogroups and haplogroup clusters in victims of non-ischaemic sudden cardiac death (*N* = 78) and in population controls (*N* = 537)

Haplogroup	SCD	SCD %	Cluster %	Controls	Controls %	Cluster %	OR (CI 95%)
H	15	19.2	56.4	119	22.2	44.7	0.84 (0.45–1.52)
H1	21	26.9		93	17.3		1.76* (1.02–3.04)
V	8	10.3		28	5.2		2.08 (0.91–4.74)
U5	19	24.4	32.1	144	26.8	33.3	0.88 (0.51–1.53)
U	4	5.1		16	3.0		1.76 (0.57–5.41)
K	2	2.6		19	3.5		0.72 (0.16–3.14)
J	3	3.8	5.1	26	4.8	10.8	0.79 (0.23–2.66)
T	1	1.3		32	6.0		0.21 (0.03–1.52)
W	5	6.4	6.4	29	5.4	9.0	1.20 (0.45–3.20)
I				9	1.7		
X				10	1.9		
Other				12	2.2	2.2	

*p < 0.05

*CI* confidence interval, *OR* odds ratio

## Discussion

We did not find m.3243A > G or other pathogenic mtDNA mutations among the 280 SCD subjects. Mutations were analysed in DNA from cardiac muscle, the clinically relevant tissue, that had been collected prospectively during a 7-year period in a defined population. In Finland, autopsy is required by law, when the death is not known to be caused by a disease or if the deceased had not been treated by a physician during the latest illness, or if the death was otherwise unexpected (Act on the Inquest into the Cause of Death 459/1973, Finnish Law). All subjects dying suddenly and unexpectedly were therefore autopsied and subjects with coronary heart disease or non-cardiac disease as the cause of death were excluded.

Mitochondrial diseases are common inherited neurological disorders, in which cardiac manifestations are among the core features [5, 28]. The incidence of major adverse cardiac events has been reported to be increased especially among patients harbouring the m.3243A > G mutation or a single mtDNA deletion [29]. Analysis of heart rate variability has revealed altered cardiac autonomic regulation in patients with m.3243A > G further indicating that regular follow-up of cardiac function is essential in these patients [29, 30]. Moreover, cardiac examination should be provided to asymptomatic mutation carriers, who might have a high heteroplasmy in the heart, and SCD might be the first disease manifestation [8, 30].

Mitochondrial cardiomyopathy may result from several other mtDNA mutations [10], such as the m.3260A > G transition in *MT-TL1* [9] causing maternally inherited cardiomyopathy. Intriguingly, the overlapping region of *MT-ATP6* and *MT-ATP8* seems to be a hotspot for mutations causing cardiomyopathy indicating that human heart is vulnerable to the dysfunction of mitochondrial ATPase [31, 32]. Cardiomyopathy has also been reported in rare cases with hearing impairment caused by m.1555A > G or Leber’s hereditary optic neuropathy caused by m.11778G > A [33, 34]. In addition, disrupted mtDNA maintenance can lead to decreased mtDNA copy number causing continuous energy deprivation and cardiomyopathy [35]. The mtDNA copy number has been shown to inversely correlate with the risk of SCD [36].

We found that superhaplogroup HV was more frequent among SCD subjects than among controls, and the difference could be attributed to 1.6-fold excess of haplogroup H1. Haplogroup H has previously been reported to be associated with dilated cardiomyopathy [37] and with a heart failure requiring heart transplant [38]. Interestingly, functional differences have been reported between haplogroups. For example, the oxygen consumption rate has been found to be decreased in haplogroup H when compared with haplogroup Uk [11]. Our results suggest that in population, haplogroup H1 may be a risk factor for non-ischaemic SCD.

Differences in ethnicity between the subjects and controls should be taken into consideration, even if both groups were ascertained from the population of Northern Ostrobothnia. The Finnish population has remained rather isolated up to the recent years and, consequently, non-European haplogroups were not found in the SCD subjects or the controls. However, there is a Saami population of 10,000 people in Finland and the frequency of mtDNA haplogroup H is exceptionally low among them [39]. In the province of Northern Ostrobothnia, there are 169 residents, whose native language is Saami (Statistics Finland; https://www.stat.fi/index_en.html) and some 850 residents who are ethnically Saami given the proportion that one out of five Saami subjects has reported Saami language as the native language. These figures suggest that the Saami make 0.21% of the population of Northern Ostrobothnia. Hence, the probability of one and only one Saami subject among the 537 controls is 0.37 suggesting that it is unlikely that possible Saami individuals among the controls would deflate the results.

Our results suggest that in the general population, mtDNA mutations are a rare cause of non-ischaemic SCD. Cardiac investigations and follow-up should be targeted to individuals harbouring pathogenic mtDNA mutations associated with cardiac complications. Identification of such individuals requires increased awareness of mitochondrial disease in the general population and among health care professionals, with genetic counselling and offering genetic testing for at-risk family members. In addition, we report an association between SCD risk and mtDNA haplogroup H1, suggesting that variation of mitochondrial energetics modifies the risk of SCD in population.

## Electronic supplementary material

ESM 1 (PDF 111 kb)

ESM 2 (PDF 276 kb)

ESM 3 (PDF 332 kb)

## Data Availability

All data generated or analysed during this study are included in this published article and its supplementary information files.

## Abbreviations

CAD: coronary artery disease
CM: cardiomyopathy
MELAS: Mitochondrial encephalomyopathy, lactic acidosis and stroke-like episodes
mtDNA: mitochondrial DNA
OXPHOS: oxidative phosphorylation
SCD: sudden cardiac death
