# Supplementary figures and images for: Mitochondrial DNA variation in sudden cardiac death: a population-based study
Source: Int J Legal Med. 2019 May 31;134(1):39–44. doi: 10.1007/s00414-019-02091-4 (PMC6949201; doi:10.1007/s00414-019-02091-4)

## Supplemental figure 1. Study subjects.

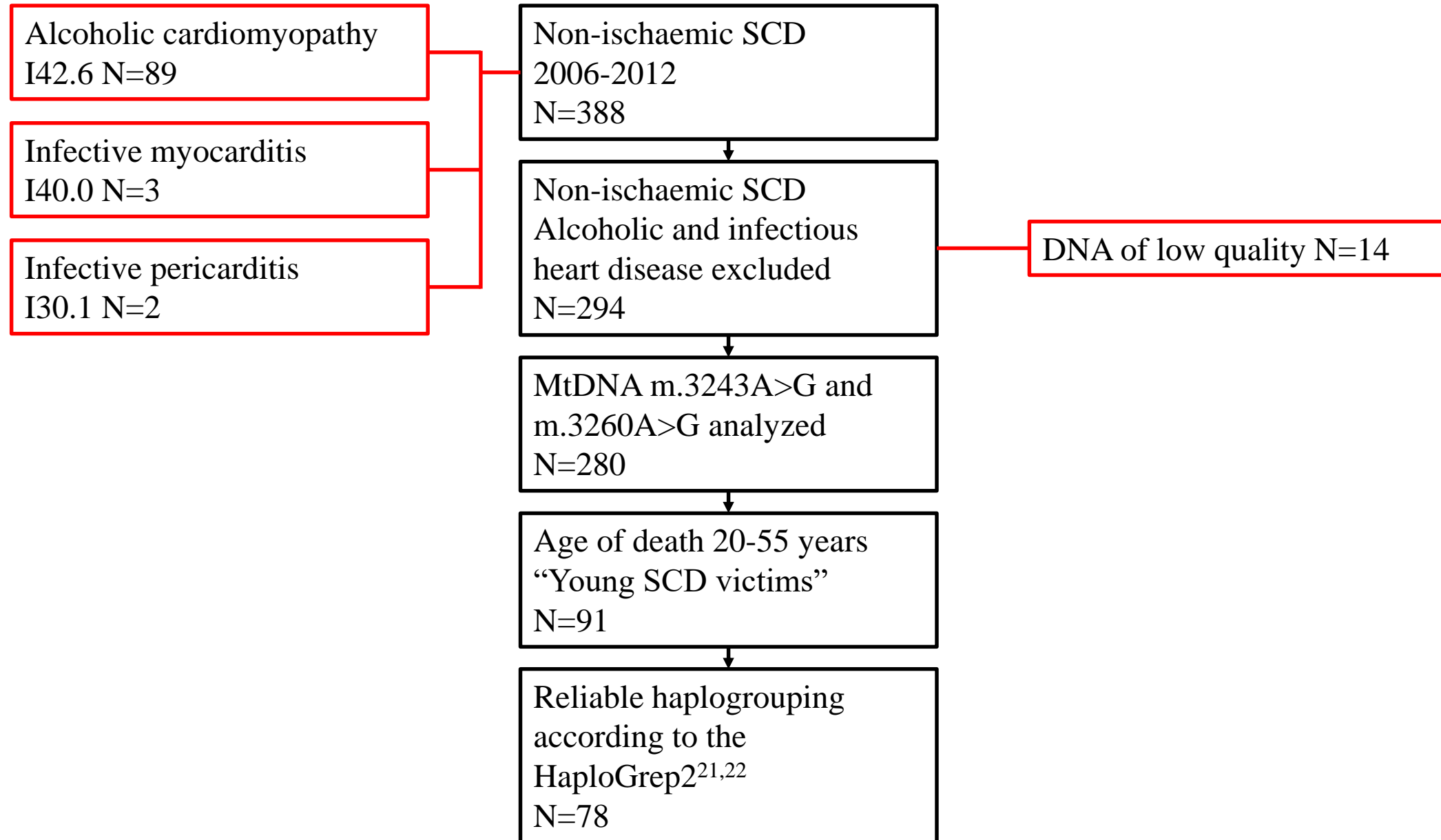

Supplement: Supplementary file 1 — (PDF 111 kb) [file 414_2019_2091_MOESM1_ESM.pdf]
