# Supplementary material for: Mitochondrial DNA variation in sudden cardiac death: a population-based study
Source: Int J Legal Med. 2019 May 31;134(1):39–44. doi: 10.1007/s00414-019-02091-4 (PMC6949201; doi:10.1007/s00414-019-02091-4)
Supplement: Supplementary file 2 — (PDF 276 kb) [file 414_2019_2091_MOESM2_ESM.pdf]

Supplementary table 1. Primers and conditions in the Piko™ Thermal Cycler (Thermo Fisher Scientific, Waltham, MA, U.S.A.). The reactions were performed in final concentrations of 1 x reaction buffer, 5 µM for each primer, 200 µM for each dinucleotide, 3 % for DMSO, 2 % Phire II Hot Start DNA polymerase and 10-30 ng of DNA. Initial denaturation was 98 C° for 30 s, cycle denaturation 98 C° for 5 s, annealing (Ta) for primer specific temperature for 5 s, cycle extension 72 C° for size-specific time (1 s per 100 bp) and final extension of 72 C° for 60 s. Thirty cycles were performed. The secondary PCR was carried out with the same conditions using PCR-product from the first reaction as a template.

Fragment	Sequence primer L 5'	Sequence primer H 5'	Ta (C°)	Product length (bp)	Extension (s)
1	GCACTTAAACACATCTCTGCCAA	GGTGAACTCACTGGAACGGG	65.4	398	4
2	CTCACATCACCCCATAAACA	GTACTTGCGCTTACTTTGTAG	57.5	683	7
3	AGAACACTACGAGCCACAGCTT	GGAGTGGGTTTGGGGCTAGG	65	560	6
4	AATGGGCTACATTTTCTACCCC	TCTTAGCTTTGGCTCTCCTTGC	63	544	6
5	AACACAAAGCACCCAACTTACAC	CTTTTAGGCCTACTATGGGTGTTAAAT	63.5	568	6
6	TTGCCCACAGAACCCTCTAAATC	GGCAGGTCAATTTCACTGGTTA	65.2	635	7
7	TGCAAAGGTAGCATAATCACTTG	CTTGGGTGGGTGTGGGTATA	63.5	634	7
8	TAACCAGTGAAATTGACCTGCC	CTTGGGTGGGTGTGGGTATA	65	559	6
9	AAGGCCTACTTCACAAAGCGCC	GAGCGATGGTGAGAGCTAAGGT	66.3	410	5
10	TCGCTGACGCCATAAAACTCTT	AGTGCGTCATATGTTGTTCCTAG	63	604	7
11	CCTATTCTTCATAGCCGAATACA	GTGATGAGTGTGCCTGCAAAG	62	610	7
12	TCAGCTAAATAAGCTATCGGGC	TTTGCGTAGCTGGGTCTGGT	64	592	6
13	CCAAATCTCTCCCTCACTAAAC	AAGGGGAGATAGGTAGGAGTAG	59.5	596	6
14	ACAATAGCCTCATCATCCCCACCAT	TGTTAACTAAGTGTTTGTGGGT	61.2	411	5
15	CCACGCTACTCCTACCTATCTC	GCGCCGAATAATAGGTATAGTG	61.2	508	6
16	TAGATTTACAGTCCAATGCTTCAC	GTAGGAGAGAGGGAGGTAAGAG	60	381	4
17	CTTTGGCAACTGACTAGTTCCC	GTCTACGTCTATTCCTACTGTAAA	59.5	670	7
18	CTCCGGAAAAAAAGAACCATTTGG	TTCGAAGCGAAGGCTTCTCAAA	67	650	7
19	GAAACATCCTATCATCTGTAGGC	TGATAGGGGAAGTAGCGTCTTG	61	383	4
20	CCATTTCATAACTTTGTCAAAGTTAA	GAAACTGTGGTTTGCTCCACAG	61	671	7
21	CCTGCGACTCCTTGACGTTG	TTTGGTGAGGGAGGTAGGTGG	69	504	6
22	GCCCCAACTAAATACTACCGTAT	GTAGGAGGCCTGCAGTAATG	61.7	667	7
23	AGCCCACTTCTTACCACAAGG	GGGACTTCTAGGGGATTTAGCG	63.5	695	7

Supplementary table 1 continued.

Fragment	Sequence primer L 5'	Sequence primer H 5'	Ta (Cº)	Product length (bp)	Extension (s)
24	CTTCGCAGGATTTTTCTGAGCC	GATATAGGGTCGAAGCCGCAC	65	708	8
25	CCGACGGCATCTACGGCTC	GATATAGGGTCGAAGCCGCAC	66.9	421	5
26	GAGTAATAAACTTCGCCTTAATTTTAATAA	GAGGATATGAGGTGTGAGCGA	61.5	502	5
27	ACCAAATGCCCCTCATTTACA	TAGGGGTCGGAGGAAAAGGTT	65.5	478	5
28	ACCACCCACAGCCTAATTATTA	GGAGTCATAAGTGGAGTCCGT	61	569	6
29	ACTACTCACTCTCACTGCCCA	AGTAGAGTTTGAAGTCCTTGAGA	59	525	6
30	GCCCACGGGCTTACATC	GGAATTAGGGAAGTCAGGGTTAG	63.1	676	7
31	ACAAGAACTGCTAACTCATGCC	AGGATTGCTTGAATGGCTGCT	65	638	7
32	CAACTGTTCATCGGCTGAGAG	ATATCTTGTTCATTGTTAAGGTTGTG	62	654	7
33	GCTTAGGCGCTATCACCACTC	GTTGAGGTCTAGGGCTGTTAGA	62	680	7
34	TAATAGATAGGGCTCAGGCGTTT	ACTGGTTGACCATTGTTT	56.5	639	7
35	GGCATAATTAAACTTTACTTCCTC	TGTTTCTGTTGAGTGTGGGTTT	60	581	6
36	GACCACACCGCTAACAATCAATACTA	GATGGGGTGGGGAGGTCGAT	66.3	261	3
37	CCCCACAAACCCCATTACTAAAC	GAATCGTGTGAGGGTGGGACT	67	661	7
38	GCCATGCACTACTCACCAGAC	GGGGAGGGGTGTTTAAGGGG	65.1	646	7
39	GCCCTCGGCTTACTTCTCTTC	ACATAGCGGTTGTTGATGGGTG	65.6	660	7
