# Supplementary material for: Mitochondrial DNA variation in sudden cardiac death: a population-based study
Source: Int J Legal Med. 2019 May 31;134(1):39–44. doi: 10.1007/s00414-019-02091-4 (PMC6949201; doi:10.1007/s00414-019-02091-4)
Supplement: Supplementary file 3 — (PDF 332 kb) [file 414_2019_2091_MOESM3_ESM.pdf]

Supplementary table 2. Polymorphisms in the mtDNA region m.577-m.16023 in Finnish subjects died from non-ischaemic sudden cardiac death (age of death ≤ 55 years).

Gene	Polymorphism	AA* change	Gene	Polymorphism	AA change
	m.709G>A			m.4613A>G	
	m.750A>G			m.4639T>C	p.Ile57Thr
	m.896A>G			m.4646T>C	
	m.958C>T			m.4732A>G	p.Asn88Ser
	m.961T>G			m.4745A>G	
MT-RNR1	m.1189T>C			m.4769A>G	
	m.1211G>A			m.4812G>A	p.Val115Ile
	m.1243C>T			m.4851C>T	
	m.1341C>T			m.4859T>C	
	m.1438A>G			m.4917A>G	p.Asn150Asp
	m.1536A>G			m.4928T>C	
	m.1721C>T			m.5004T>C	
	m.1811A>G		*MT-ND2*	m.5046G>A	p.Val193Ile
	m.1888G>A			m.5060C>T	
	m.1923C>T			m.5153A>G	
	m.2098G>A			m.5164G>A	p.Arg232His
	m.2259C>T			m.5229C>A	
MT-RNR2	m.2405.1C			m.5240A>G	
	m.2445T>C			m.5263C>T	p.Ala265Val
	m.2581A>G			m.5319A>T	p.Thr284Ala
	m.2706A>G			m.5331C>T	p.Leu288Ile
	m.2772C>T			m.5390A>G	
	m.3010G>A			m.5426T>C	
	m.3116C>T			m.5444C>T	
	m.3197T>C			m.5460G>A	p.Ala331Thr
	m.3333C>T		*MT-TA*	m.5646C>T	
	m.3363C>T		*MT-NC4*	m.5656A>G	
	m.3434A>G	p.Tyr43Cys	*MT-TC*	m.5773G>A	
	m.3480A>G			m.5780G>A	
	m.3505A>G	p.Thr67Ala	*MT-TY*	m.5840C>T	
	m.3531G>A			m.5975A>G	
	m.3549C>T			m.5984A>G	
	m.3571C>T	p.Leu89Phe		m.5999T>C	
	m.3720A>G			m.6045C>T	
MT-ND1	m.3738C>T			m.6047A>G	
	m.3742C>T			m.6152T>C	
	m.3796A>G	p.Thr164Ala		m.6221T>C	
	m.3984G>A			m.6242C>A	
	m.3992C>T	p.Thr229Met		m.6260G>A	
	m.4015C>T	p.Leu237Phe	*MT-CO1*	m.6261G>A	p.Ala120Thr
	m.4024A>G	p.Thr240Ala		m.6267G>A	p.Ala122Thr
	m.4059C>T			m.6272A>G	
	m.4065A>G			m.6314C>T	
	m.4093A>G	p.Thr263Ala		m.6357C>T	
	m.4216T>C	p.Tyr304His		m.6392T>C	
MT-TI	m.4295A>G			m.6446G>A	
MT-TM	m.4452T>C			m.6455T>C	
MT-ND2	m.4580G>A			m.6464C>A	

*Amino acid

Supplementary table 2. Polymorphisms in the mtDNA region m.577-m.16023 in Finnish subjects died from non-ischaemic sudden cardiac death (age of death ≤ 55 years).

Gene	Polymorphism	AA change	Gene	Polymorphism	AA change
MT-CO1	m.6489C>A	p.Leu196Ile	*MT-ATP6*	m.8705T>C	p.Met60Leu
	m.6497T>C			m.8720G>A	p.Gly65Ala
	m.6554C>T			m.8723G>A	p.Arg66Gln
	m.6620T>C			m.8776C>T	p.Leu84Phe
	m.6680T>C			m.8839G>A	p.Ala105Thr
	m.6752G>A			m.8842A>G	p.Ile106Val
	m.6776T>C			m.8843T>C	p.Ile106Thr
	m.6810G>A	p.Ala303Thr		m.8869A>G	p.Met115Val
	m.6884C>T			m.8898C>T	
	m.7028C>T			m.8923A>G	p.Thr133Ala
	m.7055A>G			m.8994G>A	
	m.7114C>T	p.Thr404Ile		m.9003C>A	
	m.7130C>T			m.9009C>T	
	m.7309T>C	p.Ile469Thr		m.9039G>A	
	m.7385A>G			m.9052A>G	p.Ser176Gly
	m.7444G>A	p.Ter514Lys		m.9055G>A	p.Ala177Thr
MT-CO2	m.7640G>A	p.Glu19Lys		m.9066A>G	
	m.7690C>T			m.9117T>C	
	m.7706G>A	p.Ala41Thr		m.9123G>A	
	m.7768A>G		*MT-CO3*	m.9290C>T	
	m.7805G>A	p.Val74Ile		m.9333C>T	p.Leu43Phe
	m.7859G>A	p.Asp92Asn		m.9338A>G	
	m.7864C>T			m.9365C>T	
	m.7912G>A			m.9477G>A	p.Val91Ile
	m.7918C>T			m.9488C>T	
	m.7927C>T			m.9612G>A	p.Val136Met
	m.7963A>G			m.9635A>C	
	m.8156G>A	p.Val191Leu		m.9667A>G	p.Asn154Ser
	m.8216C>T			m.9698T>C	
	m.8251G>A			m.9777G>A	p.Gly191Ser
	m.8260T>C		*MT-TG*	m.10044A>G	
	m.8269G>A		*MT-ND3*	m.10097A>G	
MT-NC7	m.8271A>T			m.10101T>C	
	m.8290delC			m.10238T>C	
MT-ATP8	m.8428C>A	p.Phe21Leu		m.10343C>T	
	m.8448T>C	p.Met28Thr		m.10398A>G	p.Thr114Ala
	m.8539C>T	p.IleMet	*MT-TR*	m.10463T>C	
	m.8541T>C	p.CysTyr	*MT-ND4/L*	m.10550A>G	
	m.8565A>G	p.Gln13Arg		m.10589G>A	
	m.8572G>A			m.10667T>C	
MT-ATP6	m.8539C>T	p.Leu5Met		m.10733C>T	
	m.8541T>C			m.10771A>G	
	m.8565A>G		*MT-ND4*	m.10877C>T	
	m.8572G>A	p.Gly16Ser		m.10927T>C	
	m.8601A>G			m.10930C>T	
	m.8614T>C			m.11016G>A	p.Ser86Asn
	m.8640C>T			m.11025T>C	p.Leu89Pro
	m.8697G>A			m.11143C>T	

Supplementary table 2. Polymorphisms in the mtDNA region m.577-m.16023 in Finnish subjects died from non-ischaemic sudden cardiac death (age of death ≤ 55 years).

Gene	Polymorphism	AA change	Gene	Polymorphism	AA change
MT-ND4L	m.11197C>T		*MT-ND5*	m.13395A>G	
	m.11251A>G			m.13401T>C	
	m.11263C>T			m.13617T>C	
	m.11299T>C			m.13637A>G	p.Gln434Arg
	m.11332C>T			m.13680C>T	
	m.11377G>A			m.13681A>G	p.Thr449Ala
	m.11428C>T			m.13708G>A	p.Ala458Thr
	m.11467A>G			m.13718G>C	p.Ser461Thr
	m.11485T>C			m.13740T>C	
	m.11542C>T			m.13759G>A	p.Ala475Thr
	m.11560A>G		*MT-ND6*	m.14167C>T	
	m.11674C>T			m.14180T>C	p.Tyr165Cys
	m.11719G>A			m.14182T>C	
	m.11732T>C			m.14233A>G	
	m.11812A>G			m.14325T>C	p.Asn117Asp
	m.11840C>T			m.14356C>T	
	m.11899T>C			m.14365C>T	
	m.11914G>A			m.14582A>G	p.Val31Ala
	m.11947A>G		*MT-CYB*	m.14766C>T	p.Thr7Ile
	m.11953C>T			m.14793A>G	p.His16Arg
	m.11969G>A	p.Ala404Thr		m.14798T>C	p.Phe18Leu
	m.12308A>G			m.14848C>A	
	m.12127G>A			m.14867G>A	p.Leu41Met
MT-TH	m.12192G>A			m.14875C>A	
MT-TL2	m.12308A>G			m.14905G>A	
MT-ND5	m.12363C>T			m.15028C>A	
	m.12366A>G			m.15043G>A	
	m.12372G>A			m.15058C>T	
	m.12414T>C			m.15074T>C	p.Ser110Pro
	m.12474C>T			m.15218A>G	p.Thr158Ala
	m.12501G>A			m.15250C>T	
	m.12540A>G			m.15254G>A	p.Val170Met
	m.12612A>G			m.15280C>T	
	m.12613G>A	p.Ala93Thr		m.15430C>T	
	m.12618G>A			m.15452C>A	p.Leu236Ile
	m.12630G>A			m.15466G>A	
	m.12669C>T			m.15498G>A	p.Gly251Asp
	m.12705C>T			m.15511T>C	
	m.12846G>A			m.15607A>G	
	m.12997G>A	p.Ala221Thr		m.15618T>C	p.Val291Ala
	m.13002C>T			m.15693T>C	p.Met316Thr
	m.13135G>A	p.Ala267Thr		m.15773G>A	p.Val343Met
	m.13145G>A	p.Ser270Asn		m.15784T>C	
	m.13152A>T			m.15884G>C	p.Ala380Pro
	m.13326T>C		*MT-TT*	m.15904C>T	
	m.13350A>G			m.15924A>G	
	m.13362C>T			m.15928G>A	
	m.13368G>A			m.15940T>C	
